# Human neural stem cells drug product: Microsatellite instability analysis

**DOI:** 10.1371/journal.pone.0273679

**Published:** 2022-08-30

**Authors:** Valentina Grespi, Cecilia Caprera, Claudia Ricciolini, Ilaria Bicchi, Gianmarco Muzi, Matteo Corsi, Stefano Ascani, Angelo Luigi Vescovi, Maurizio Gelati

**Affiliations:** 1 S. Maria Hospital Terni, Terni, Italy; 2 IRCCS Casa Sollievo della Sofferenza, San Giovanni Rotondo, Italy; International Medical University, MALAYSIA

## Abstract

**Introduction:**

In central nervous system neurodegenerative disorders, stem cell-based therapies should be considered as a promising therapeutic approach. The safe use of human Neural Stem Cells (hNSCs) for the treatment of several neurological diseases is currently under evaluation of phase I/II clinical trials. Clinical application of hNSCs require the development of GMP standardized protocols capable of generating high quantities of reproducible and well characterized stem cells bearing stable functional and genetic properties.

**Aim:**

The aim of this study was to evaluate possible instabilities or modifications of the microsatellite loci in different culture passages because high culture passages represent an in vitro replicative stress leading to senescence.

Experimental method: The hNSCs were characterized at different culture time points, from passage 2 to passage 25, by genetic typing at ten microsatellite loci.

**Conclusion:**

We showed that genetic stability at microsatellite loci is maintained by the cells even at high passages adding a further demonstration of the safety of our hNSCs GMP culture method.

## Introduction

In the recent years, cell-based regenerative medicine has been witnessing an unprecedented growth, as denoted by the increase of cell therapy products entering the clinical stage [1]. The clinical use of human Stem Cells (SCs) raises the fundamental question about their safety, confirming that safety of SC based therapies is crucial for their effective clinical translation [2].

Between 2012 and 2020, our group conducted two phase I clinical trials focused on Amyotrophic Lateral Sclerosis (ALS, NCT01640067) and Secondary Progressive Multiple Sclerosis (SPMS, NCT03282760), treating 18 and 15 patients, respectively, with human Neural Stem Cells (hNSCs).

The process to obtain and expand hNSCs requires extensive cell manipulation, and, in order to proceed with translation to clinical practice, the adoption of robust production and quality assurance protocols in compliance with the Good Manufacturing Practice regulations is required. In particular, the Article n. 3.3.2.3 of the European Commission Directive 2009/120/EC establishes which relevant information is needed in terms of identity, purity (e.g., adventitious microbial agents and cellular contaminants), viability, potency, karyology, tumorigenicity and suitability for the intended medicinal use [3]. The article also specifies that “the genetic stability of the cells shall be demonstrated”. This aspect is recognized as one of the major hurdles in the field of stem cell-based therapies; nevertheless, the available guidelines do not identify exclusive tests for assessing long term genetic stability. The vast majority of techniques fails in detecting alterations affecting less than 10% of the analyzed cell population, and this represents an important issue for the early detection of newly occurring somatic mutations [2] that could lead to a tumorigenic transformation. Moreover, cell transformation is associated not only to chromosomal anomalies, such as gross deletions, duplications and aneuploidies, but also to micro-deletions and micro-duplications and mutations at the single base level. Consequently, it seems appropriate to extend the analysis of genetic alterations acquired in long term culture to small molecular defects since there is no reason to assume that small genetic changes are irrelevant [2].

Each cell batch used in our studies was evaluated for genetic stability by conventional karyotype (G banding) and single nucleotide polymorphism (SNP) array, and the cells derived from each donor were tested in a nude mice model to verify the tumorigenic potential *in vivo* [4]. In order to improve the characterization of the hNSC-based drug and the critical aspect of genetic stability, the aim of this study was to evaluate the microsatellite instability (MSI) to assess possible genetic damage in the hNSCs and their tumoral drift in relation to *in vitro* expansion and cellular senescence.

Microsatellites are DNA sequences composed of short nucleotide segments (1–10 nucleotides, also known as short tandem repeats (STRs)) which repeat sequentially. Microsatellites are highly polymorphic and the length of the microsatellite locus have a high mutation rate [5]. Due to these characteristics, as suggested by the American Type Culture Collection (ATCC) procedures, STRs can be used both for the identification and characterization of human cell lines [6].

The STR analysis is a universally accepted method for human cell line identification. STRs selected for cell line authentication are chosen because they display the highest possible variation for discriminating among human cell lines [7]. STR DNA profile analysis involves the simultaneous amplification of 17 STR markers plus amelogenin for gender determination and is capable of discriminating among human cell lines at about 1 x 10^−18^ [6].

Due to their repetitive nature, during DNA replication those sequences may expand or shrink due to strand slippage. Those errors are often recognized and repaired by the DNA MisMatch Repair (MMR) machinery. However, when MMR enzymes are defective, replication errors will evade repair and persist in somatic cells, and this is referred to as MSI. MSI is an important indicator of larger genome instability and has been identified in over 20 cancer types. MSI is observed in 15% of sporadic colorectal tumors diagnosed in the United States, and it has been reported in glioblastomas, lymphomas, stomach, urinary tract, ovarian and endometrial tumors [5].

The MMR system is the main post-replicative DNA repair pathway, which guarantees genomic stability in actively proliferating cells. Mutations in MMR genes lead to MMR deficiency (dMMR). DNA dMMR is one of the most important mechanisms leading to underlying genomic instability, chromosomal instability, and MSI. DNA MMR-related proteins consist at least of seven types, including h-MLH1, h-MLH3, h-MSH2, h-MSH3, h-MSH6, h-PMS1 and h-PMS2. Functional heterodimers are formed by specific combination between MMR-related proteins to recognize mismatch base pairs [8]. However, when DNA MMR-related genes experience mutation or epigenetic changes, these genes lose their ability to synthesize MMR-related proteins, thus resulting in DNA MMR deficiency and MSI occurrence [9]. DNA methylation is the best characterized epigenetic modification. Methylation may impact gene transcription by direct interference with transcription factors or with methyl-CpG-binding proteins that modify histones and thereby inactivate the respective promoter region. An increase in the methylation status of several gene promoters with age is well documented and, among them, some MMR genes were also found to be hypermethylated with a related inhibition of their expression. Furthermore, it has been proposed that epigenetic changes, such as DNA methylation, play a central role in senescence and aging and that methylation levels gradually decrease upon long-term culture [10].

The aim of this study was to evaluate possible instabilities or modifications of the microsatellite loci in different culture passages that represent an *in vitro* imposed replicative stress. Here, we show the evaluation of genetic stability upon *in vitro* hNSCs culture by analysis, used as a biosafety and surrogate marker of molecular integrity.

## Materials and methods

### Human neural stem cells

The hNSCs used in this study have been produced and characterized in the Cell Factory of the Santa Maria Hospital (Terni, Italy), authorized by the Italian Medicines Agency (AIFA) for the production of hNSCs to be used for clinical trials. The methodology applied to isolate, expand, characterize and cryopreserve the lines is based on the Neurosphere assay [4]. In order to produce hNSCS according to GMP standard, a panel of cellular, functional, and biochemical analyses must be assessed, which include, but are not limited to, karyotype analysis, stable differentiation and growth capacity, and lack of biological contamination by adventitious agents [11]. The collection of the fetal neural tissues was authorized by the Ethical Committee of the “Fondazione IRCCS Casa Sollievo della Sofferenza” research hospital (Prot. N. 01/CE 25-01-2012). Each donor provided written informed consent to the tissue collection only after the fetus death.

The hNSCs used in this work, were obtained from fetal brain tissue derived from fetuses that underwent miscarriage or natural in utero death. The entire production process, starting from tissue procurement to cellular drug release passing through a cryopreservation step, is compliant to the above mentioned criteria.

For this study, six cryopreserved hNSCs lines, derived from six different fetuses, were thawed at different passages (Table 2 shows for each line the earlier and the later passage) and cultured or a further step. The hNSCs were collected by centrifugation and fixed with formalin (we used formalin 10% for 10 minutes) and embedded into paraffin to produce formalin-fixed paraffin-embedded (FFPE) blocks used routinely by our pathological anatomy unit in the MSI examination as explained into following paragraphs.

### Isolation of genomic DNA

Serial 8-μm sections of FFPE hNSCs blocks were prepared using DNA histology precautions. The blocks of each case of hNSCs were dissected on unstained slides. Microdissection of the unstained slides was performed in a laminar flow hood after UV irradiation. Only paraffin samples with an hNSCs content of at least 25% were included in the test. MSI assays were performed on microdissected DNA, extracted using the "MagCore Genomic DNA FFPE One-Step" Kit (Diatech Pharmacogenet-ics, CE-IVD) following the manufacturer’s instructions using MagCore Automated Nucleic Acid Extractor Super (RBCBioscience). Microvolume samples were quantified by a spectrophotometer system "NanoDrop One Microvolume UV-Vis Spectrophotometer" (Thermo Fisher Scientific). Purified nucleic acid was quantified by spectrophotometric determination at 260 nm (NanoDrop One, Thermo Scientific). Furthermore, the quantification of the DNA ensured an accurate and precise measurement of the amount of DNA actually amplifiable in the subsequent multiplex PCR reaction and an assessment of the degree of DNA fragmentation.

### Short tandem repeat analysis

The analysis of STRs was carried out using the "Titano MSI" kit (Diatech Pharmacogenetics, CE-IVD), used in clinical practice for the determination of MSI status in colorectal cancer, especially in the hereditary non-polyposis colorectal cancer. Briefly, the extracted DNA was analyzed with the MSI Titano kit by multiplex amplification PCR with fluorescent primers following the manufacturer’s instructions. The Titano MSI kit allows the determination of the microsatellite instability status by multiplex amplification with fluorescent primers and subsequent DNA fragment analysis on an automated sequencer. Starting from 80 ng of the extracted DNA, this tool is able to detect variations in the length of 10 different microsatellite loci (see Table 1) by comparing peak profiles generated from the capillary electrophoresis (SeqStudio Genetic Analyzer, AppliedBiosystems by Thermo Fischer Scientific, CE-IVD). The microsatellite status of each sample was determined based on the percentage of unstable loci. Status was defined as MSI-high (MSI-H) when 4 or more markers displayed instability and MSI-low (MSI-L) when 1 to 3 markers exhibited instability. A sample was classified as microsatellite stable (MSS) when no MSI was found.

**Table 1:** The different microsatellite loci analyzed.

	Locus	Repetitions	Length^1^ (bp)
**BETHESDA panel**	BAT25	(T)_25_	110–115
	BAT26	(A)_26_	105–120
	D2S123	(CA)_n_	190–215
	D17S250	(CA)_n_	140–185
	D5S346	(CA)_n_	100–120
	BAT40	(A)_40_	97–125
	D18S58	(CA)_n_	145–165
	NR21	(T)_21_	96–112
	NR24	(T)_24_	120–140
	TGFβRII	(A)_n_	78–86
	TPOX	(TGAA)_n_	225–250
	TH01	(TCAT)_n_	155–165

^1^ The range is indicative and reflects the most frequent alleles.

^2^ TPOX and TH01 are control markers used to identify sample exchanges or contaminations.

## Results

The hNSCs were characterized at different culture time points, from passage 2 to passage 25, by genetic typing at ten microsatellite loci (see Table 2). As shown in Table 2, samples were microsatellite stable and allele patterns were maintained all over the culture period for all analyzed hNSCs lines, thus indicating that, in these cellular cultures, repeated replications *in vitro* did not alter genetic stability in simple sequence repeats.

**Table 2:** Results for the hNSCs lines.

hNSC lines	Cell Passage			BAT25	BAT26	D2S123	D17S250	D5S346	BAT40	D18S58	NR21	NR24	TGFβRII	MSI Analysis^1^
	Low	High												
02/13B	2		25	Stable	Stable	Stable	Stable	Stable	Stable	Stable	Stable	Stable	Stable	MSS
05/08B	5		21	Stable	Stable	Stable	Stable	Stable	Stable	Stable	Stable	Stable	Stable	MSS
05/12B	3		18	Stable	Stable	Stable	Stable	Stable	Stable	Stable	Stable	Stable	Stable	MSS
06/12B	3		21	Stable	Stable	Stable	Stable	Stable	Stable	Stable	Stable	Stable	Stable	MSS
08/12B	2		19	Stable	Stable	Stable	Stable	Stable	Stable	Stable	Stable	Stable	Stable	MSS
03/14B	4		19	Stable	Stable	Stable	Stable	Stable	Stable	Stable	Stable	Stable	Stable	MSS
PC^2^	-		-	Unstable	Unstable	Unstable	Unstable	Unstable	Unstable	Unstable	Unstable	Unstable	Stable	MSI-H

^1^ Three levels of MSI can be identified: high level MSI (MSI-H), defined as MSI in more than 4 of the standard markers; low level MSI (MSI-L), when changes exhibited from 1 to 3 of the markers and microsatellite table (MSS) in the absence of any microsatellite alterations

^2^ High level MSI was identified in a colorectal tumor sample used as test positive control.

## Discussion

Confirming the safety of cell therapy products is of paramount importance for their effective clinical translation. In particular, genetic stability and senescence appear as critical aspects to be carefully evaluated to avoid failures or safety risks.

*Ex vivo* expansion is required to obtain the sufficient number of cells to administer, but this procedure entails some risks related to genetic stability, senescence and transformation. Multiple replication *in vitro* exposes cells to the risks of accumulating genetic and epigenetic alterations, with a detrimental effect on cell biology and therapeutic properties, with the promotion of cell senescence and potential transformation [2].

MSI is a form of genome instability commonly found in cancer in which there is an accumulation of slippage mutations within STRs during DNA replication, due to the loss of the post-replication MMR function. This loss of function is generally due to genetic or epigenetic inactivation of MMR genes, such as h-MLH1, h-MSH2, h-MSH6 and h-PMS2 [12]. In cancer, these mutations often affect homopolymeric sequences located in genes involved, for instance, in cell growth or in cell death. Mutation of these tracks leads to gene inactivation and may confer a growth advantage to the cells. Several studies indicate that epigenetic modifications, such as DNA methylation, are involved in replicative senescence upon long-term culture of mesenchymal stromal cells supporting the perception that replicative senescence and aging are not only due to stochastic accumulation of cellular defects but rather represent a developmental program [10].

In this study, we addressed the impact of long-term *in vitro* culturing of human neural stem cells on simple sequence repeat stability as markers of MisMatch Repair deficiency. Different lines of hNSCs showed a stable microsatellite profile throughout the culture period comparing early passages with later passages, as further confirmation of their genetic stability. As stated above, in our GMP hNSCs production method, cellular passages of propagation and expansion are routinely repeated up to 20 times before the cells are suspended into physiological buffer and supplied to the surgery site on the day of transplantation.

In this work, we employed the “Titano MSI” kit used in clinical laboratory routine practice for the evaluation of the MSI status. This system is based on two main panels: (i) five microsatellites comprising two mononucleotide repeats (BAT25 and BAT26) and three dinucleotide repeats (D5S346, D2S123 and D17S250), i.e., the Bethesda panel, and (ii) five mononucleotide repeats (BAT25, BAT26, NR21, NR24 and NR27) [13]. Mononucleotide tracts were found to be the most sensitive markers of MSI, and further panels comprising different mononucleotide repeats have been proposed for MSI analysis. These mononucleotide markers are defined quasimonomorphic because, although they are polymorphic in the population due to the existence of variable numbers of repeat units, interallelic size differences are very small for each single locus. This property allows easy and reliable detection of MSI without the need to investigate constitutional DNA from the same patient for comparison [14]. In our future perspectives, we envisage the employment of kits that use this type of approach to evaluate the microsatellite genetic stability in hNSCs.

Furthermore, we would establish the STR profile for each cell line. This baseline STR profile will be used to create a reference database and compare it with all subsequent STR profiles performed on the various cell batches. STR DNA profiling for cell line authentication, in addition to other quality controls, can greatly improve detection of cell cross-contamination or misidentification.

## Conclusion

An essential prerequisite for the use of hNSCs in clinical trials is a comprehensive and standardized characterization of each cell culture with a particular focus on safety. The use of neural stem cells exposed to long-term expansion protocols requires the improvement of reliable tests aimed at constantly monitoring their morphological, functional and molecular characteristics to ensure the identity and safety of their use in clinical practice. As per the European Pharmacopoeia requirements, the cells used in these clinical trials were analyzed in order to confirm their microbiological safety; each batch was also tested to assess identity, potency and safety, through morphological and functional assays. Pre-clinical, clinical and in vitro non-clinical data has proved that our cells are safe, stable, and the production process can provide a high level of reproducibility of the cultures [4]. Here, we added demonstration on genetic stability at microsatellite loci is maintained by the cells even at high passages (up to 25).

Long term cultivation seems to have no consequences on hNSCs’ microsatellite stability; however, replicative senescence activation in hNSCs is a fundamental biosafety aspect. This process encompasses the permanent arrest of cell proliferation, altered morphology, changes in cellular metabolism, epigenetic regulation and protein expression. Senescent cells secrete a characteristic cocktail of factors, called SASPs (Senescence Associated Secretory Phenotype), composed of proinflammatory cytokines, growth factors and proteases. The SASP favors the infiltration of immune cells for the clearance of senescent cells, but it can also promote the establishment of an inflammatory status that alters the functionality and structure of the tissue, even creating a protumorigenic environment. To avoid transplantation of the hNSCs population enriched in senescent cells, we allow the clinical use of hNSCs spanning between the tenth and twentieth amplification cycle, based on the stability of the functional properties of hNSCs within this range of passages, but it appears therefore of paramount importance to implement the next step of improvement of our quality control in the monitoring of senescence by defining a panel of assays to investigate and attest to the onset of replicative senescence in hNSCs lines.
